# Sunflower Bark Extract as a Biostimulant Suppresses Reactive Oxygen Species in Salt-Stressed Arabidopsis

**DOI:** 10.3389/fpls.2022.837441

**Published:** 2022-07-01

**Authors:** Jing Li, Philippe Evon, Stéphane Ballas, Hoang Khai Trinh, Lin Xu, Christof Van Poucke, Bart Van Droogenbroeck, Pierfrancesco Motti, Sven Mangelinckx, Aldana Ramirez, Thijs Van Gerrewey, Danny Geelen

**Affiliations:** ^1^HortiCell, Department of Plants and Crops, Faculty of Bioscience Engineering, Ghent University, Ghent, Belgium; ^2^Laboratoire de Chimie Agro-Industrielle, Université de Toulouse, Institut National de Recherche pour l’Agriculture, l’Alimentation et l’Environnement (INRAE), École Nationale Supérieure des Ingénieurs en Arts Chimiques et Technologiques (ENSIACET), Toulouse, France; ^3^Ovalie Innovation, Auch, France; ^4^Biotechnology Research and Development Institute (BiRDI), Can Tho University, Can Tho, Vietnam; ^5^Flanders Research Institute for Agriculture, Fisheries and Food (ILVO), Melle, Belgium; ^6^SynBioC, Department of Green Chemistry and Technology, Faculty of Bioscience Engineering, Ghent University, Ghent, Belgium

**Keywords:** *Helianthus annuus*, plant extract, biostimulant, *in vitro* assay, salt stress, antioxidant

## Abstract

A survey of plant-based wastes identified sunflower (*Helianthus annuus*) bark extract (SBE), produced via twin-screw extrusion, as a potential biostimulant. The addition of SBE to Arabidopsis (*Arabidopsis thaliana*) seedlings cultured *in vitro* showed a dose-dependent response, with high concentrations causing severe growth inhibition. However, when priming seeds with SBE, a small but significant increase in leaf area was observed at a dose of 0.5 g of lyophilized powder per liter. This optimal concentration of SBE in the culturing medium alleviated the growth inhibition caused by 100 mM NaCl. The recovery in shoot growth was accompanied by a pronounced increase in photosynthetic pigment levels and a stabilization of osmotic homeostasis. SBE-primed leaf discs also showed a similar protective effect. SBE mitigated salt stress by reducing the production of reactive oxygen species (ROS) (e.g., hydrogen peroxide) by about 30% and developing more expanded true leaves. This reduction in ROS levels was due to the presence of antioxidative agents in SBE and by activating ROS-eliminating enzymes. Polyphenols, carbohydrates, proteins, and other bioactive compounds detected in SBE may have contributed to the cellular redox homeostasis in salt-stressed plants, thus promoting early leaf development by relieving shoot apical meristem arrest. Sunflower stalks from which SBE is prepared can therefore potentially be valorized as a source to produce biostimulants for improving salt stress tolerance in crops.

## Introduction

Substantial losses in biomass accompany crop production and downstream processing because of inadequate harvesting methods and a lack of valorization of by-products (Parfitt et al., 2010). To reduce the ecological footprint of agricultural practices, the Food and Agriculture Organization of the United Nations (FAO) identified two primary targets: “agricultural sustainability” and “global food losses” (FAO, 2021). In view of these targets, we urgently need to transform agricultural waste into value-added products. Crop waste is a natural resource for refining and recovering bioactive ingredients (Van Tang, 2017). Indeed, various molecules are abundant in unused biomass, some of which can be developed as plant biostimulants (PBs) (Xu and Geelen, 2018; Huang et al., 2021). The development and commercialization of PBs is a rapidly growing business, estimated at USD 3.2 billion in 2021 with a projected compound annual growth rate (CAGR) of 12.1% (Markets and Markets, 2021). Strikingly, plant extract-based PBs exhibited the highest effectiveness in yield enhancement of field crops (Li et al., 2022). Compared with synthetic chemical additives for crop improvement, PBs derived from natural resources like plant byproducts are poised to encompass a lower environmental risk and impact (Kumar et al., 2019). PBs are more likely to pass the regulatory restriction of fertilizers from natural origins imposed by legislation (Regulation [EU], 2019).

The main methods of PBs application are foliar spraying, seed priming, and soil drenching (Vertified Market Research, 2021). Hence, most primary screening assays are designed to screen putative biostimulant activity starting from seed germination and the growth responses of seedlings (García-García et al., 2020). The monitoring of seedling growth allows for *in vitro* assays under controlled conditions, short evaluation periods, and assessment of a broad spectrum of responses (Colegate and Molyneux, 2007). Subsequent bioassays are dedicated to monitoring specific plant responses. For example, seed priming tests report the effect of chemical reagents on seed germination and early seedling development (Lutts et al., 2016). Arabidopsis (*Arabidopsis thaliana*) seedlings grown *in vitro* are widely used to study the effects of exogenous chemicals on root and shoot growth (Trinh et al., 2018). An alternative to growth assays is measuring the longevity of mature leaf discs punched from mature leaves that normally senescence in a matter of days (Chiu et al., 2021). *In vitro* bioassays also allow for the quantitative impact of stress responses, and the combining of the results from multiple assays provides a reasonable indication for possible biostimulant activity under field conditions.

Plant-based raw materials are typically rich in diverse metabolites (Zulfiqar et al., 2020). Various plant extracts have also improved stress tolerance, often attributed to antioxidants (De Diego and Spíchal, 2020). Polyphenols, abundant in many plant extracts, are a class of bioactive antioxidants that scavenge *in vitro* and *in vivo* reactive oxygen species (ROS) (Stagos, 2020). For instance, many polyphenols are found in various bark by-products from woody species such as oak and willow (Dróżdż and Pyrzynska, 2017; Dou et al., 2018; Tanase et al., 2019). Sunflower (*Helianthus annuus*) seeds and florets are also rich in polyphenols with antioxidant activity (Karamać et al., 2012; Ye et al., 2015). The trichomes isolated from the surface of sunflower stems contain many flavonoids, which are typically showing antioxidant activity (Brentan Silva et al., 2017).

Sunflower is an annual crop produced for its seed and is the fourth most important oilseed crop responsible for 10% of the world’s edible plant-derived oil (Dantas et al., 2017). The leaves and stems are usually not harvested and left on the field as organic compost. The worldwide production of sunflower foliage and stems is an estimated 15.2 megatons per year (United Nations Industrial Development, 2007). Because of this substantial amount of biomass, stem material is considered a source of fiber used in biocomposite panels and other fiber-rich materials. The stalks are separated into the bark (external “woody” part, 90% w/w), which is rich in lignocellulose, and the pith (internal part, 10% w/w) (Evon et al., 2018; Verdier et al., 2020). In addition to fiber, the stalks can potentially be refined through the advanced twin-screw extrusion technology and used for various added-value applications in the agrochemical industry.

This study shows that (1) sunflower bark extract (SBE) can be produced as a side stream during twin-screw extrusion of fiber from stems; (2) SBE is a complex mixture of water-soluble molecules, several of which have bioactivity on plant growth; (3) exogenous application of SBE mitigates salt stress-induced growth inhibition of *in vitro* grown Arabidopsis.

## Materials and Methods

### Sunflower Stalk Collection and Bark Extraction

Sunflower stalks were collected with a forage harvester with the assistance of Ovalie Innovation in Autumn 2018 (Samaran, Gers department, southwest France). The stalks were stored in a ventilated box and dried with ventilated air at 40°C for 24 h. The bark was mechanically separated from the pith using a three-step procedure: (1) grinding of stalks using a hammer mill (Electra Goulu N, France) fitted with a 32 mm sieve; (2) de-dusting of the ground material using a vibrating sieve shaker (Ritec 600, France) equipped with a 1 mm screen; (3) aspiration of pith particles. Pith and bark particles were separated based on their differential density (i.e., 30 and 140 kg/m^3^, respectively).

Here, the twin-screw extrusion technology was used as an innovative technique for the thermo-mechanical and organic solvent-free extraction of biomolecules (Evon et al., 2018; Vandenbossche et al., 2019). The bark was then fractionated into a pulp and a liquid extract made of water-soluble compounds using a co-rotating and co-penetrating twin-screw extruder (Clextral Evolum HT 53, France). The extruder barrel (1.9 m in length) consisted of eight modules, each 4D in length (D is the screw diameter, i.e., 53 mm), except for module 1, which had an 8D length. A filter section consisting of six hemispherical dishes with 1 mm diameter perforations outfitted on module 7 enabled filtrate collection. During the liquid/solid fractionation process, bark with 10.0 ± 0.1% moisture content was fed at the level of module 1 using a gravimetric feeder. Water was injected at the end of module 2 using a piston pump at a liquid/solid ratio of 2.9 (i.e., 10.2 and 29.6 kg/h for the inlet flow rates of bark and water, respectively). For optimal operation, a specific screw configuration was applied. Bilobe paddles (BL22) were positioned in module 5 to favor strong mixing between the liquid and the solid. In addition, reversed pitch screws (CF2C) were positioned in module 8, immediately downstream from the filtering sieves to separate the liquid (i.e., the filtrate) and solid (i.e., the pulp) phases continuously by compression action. The temperature was set at 80°C in module 2, at 100°C along the extracting zone (modules 3–6), and 110°C in the pressing part (module 8). The rotation speed of the screws was set at 250 rpm.

The filtrate collected at the bottom of the filtration module (module 7) was centrifuged to remove the small solid particles and driven through the filter. Then, the clarified filtrate was concentrated by partial water evaporation and freeze-dried, producing SBE as a powder product stored in the dark at 4°C until use.

### Chemical Characterization of Starting Sunflower Bark and Sunflower Bark Extract

The moisture and dry matter contents of solids in the starting sunflower bark were determined according to ISO 665:2000 (ISO, 2000). The mineral content of starting bark was quantified according to ISO 749:1977 (ISO, 1977), and lipids were assessed according to ISO 659:2009 (ISO, 2009). Cell wall polymers, including cellulose, hemicellulose, and lignin, were quantified using the ADF–NDF method (ADF for acid detergent fiber, and NDF for neutral detergent fiber) of Van Soest and Wine (1967, 1968). After 1 h of boiling in water, water-soluble compounds were calculated from biomass losses.

Inside the SBE, the soluble protein and digestible carbohydrate contents were analyzed by colorimetric methods (Deans et al., 2018). Total phenolics content (TPC) was estimated by the Folin–Ciocalteu method (Sánchez-Rangel et al., 2013). The total flavonoid content (TFC) was measured following two aluminum complexation methods using quercetin and rutin as reference flavonoids (Pękal and Pyrzynska, 2014). The total *in vitro* antioxidant capacity (TAC) of SBE was determined using the 2,2-diphenyl-1-picrylhydrazyl (DPPH) and the 2,2′-azino-bis (3-ethylbenzothiazoline-6-sulfonic acid) (ABTS) assay (Xiao et al., 2020). Trolox (TE) was used as a standard antioxidant to calculate the equivalent antioxidant capacity of samples. The DPPH assay was slightly modified (Xiao et al., 2020). A total of 20 μl sample was added with 100 μl 200 mM DPPH and 80 μl 50 mM Tris–HCl buffer (pH 7.4).

#### Chemical Profiling of Sunflower Bark Extract Using UHPLC-PDA-High-Resolution Mass Spectrometer Analysis

Sunflower bark extract was dissolved in water at 1 mg/mL and pushed through a 0.22 μm filter (Millix-GV, Millipore). Samples were subjected to ultra-high-performance liquid chromatography (Acquity UPLC) coupled to a PDA detector (UPLC eLambda 800 nm) and a SYNAPT G2-S High-Resolution Mass Spectrometer (HRMS) (Waters, Milford, MA, United States). Prepared samples were chromatographically separated on an ACQUITY UPLC BEH C18 column (1.7 μm, 2.1 mm × 150 mm) protected by an ACQUITY UPLC BEH C18 VanGuard Precolumn (1.7 μm, 2.1 mm × 5 mm) (Waters). The mobile phase A was 0.1% formic acid in water (solvent A) and the mobile phase B was 0.1% formic acid in acetonitrile (solvent B) at a flow rate of 0.35 mL/min with following gradient: 95% A–5% B (0–18 min), 100% B (18–25 min), 95% A–5% B (25.1–30 min). PDA data was recorded between 220 and 550 nm. Ions were detected in the positive electrospray ionization (ESI+) and negative electrospray ionization (ESI−) modes. The ESI conditions were set as follows: capillary voltage of 3.0 kV (ESI+)/2.0 kV (ESI−), source temperature of 120°C, cone voltage of 30 V (ESI+)/40 V (ESI−), and desolvation temperature of 500°C with a desolvation gas flow of 800 L/h. The collision-induced dissociation (CID) was set at 4 eV for precursor ion, and MS/MS fragment ion information was obtained with a collision energy ramp from 8 to 40 eV. The HRMS was calibrated between 50 and 1200 Da with a sodium formate solution prior to analysis. The injection volume was set at 5 μl, and each sample was analyzed in duplicate. All data were recorded at resolution mode (20,000 FWHM) in centroid full scan MS*^e^* mode (data-independent acquisition, DIA). A 200 pg/μl leucine enkephalin solution was continuously infused during analysis to perform lockmass correction (m/z 556.2771 in positive ion mode and m/z 554.2615 in negative ion mode) during analysis. Blanks containing only the mobile phase without any sample were injected between each batch of samples.

To determine the chemical composition of SBE, the raw MS/MS data were processed for feature detection and alignment with MS-DIAL software version 4.48 (Blaženović et al., 2017). The detailed settings are listed in Supplementary Table 1, and are adapted from Lee et al. (2019). To gain a high confidence in the peak identification, only the features present in both runs from the same sample were considered for alignment correction. Next, aligned features meeting the criteria were selected with a total weighted similarity score of over 60 (overall library-matching score based on retention time, accurate mass, isotope ratio, and MS^E^ spectra) (Tsugawa et al., 2015). Then, we searched these chosen features for further compound prediction in MS-FINDER software version 3.50 (Tsugawa et al., 2016) among online metabolites databases, including PlantCyc (plant), KNApSAcK (natural product), FoodDB (Food), ChEBI (Biomolecules), and PubChem (Biomolecules), with the agreement of identification confidence levels (level 3) (Schymanski et al., 2014). In each feature, the predicted formula and structure with the top total score were reported as the final candidate compound (Tsugawa et al., 2019). Finally, the identified compounds were assigned taxonomy based on their chemical characterization represented as InChIKey (International Chemical Identifier) in ChemOnt ontology via ClassyFire (Djoumbou Feunang et al., 2016).

#### Pesticide Residue Detection in Sunflower Bark Extract

Lyophilized powder of SBE was analyzed for the presence of over 500 pesticide residues (Regulation [EC], 2005, 2009) (Primoris, Belgium). Briefly, the pesticide(s) were extracted from the crude sunflower bark material with acidified acetonitrile (QuEChERS extraction) or acetonitrile with 0.5% acetic acid. Pesticides were quantitatively determined using LC–MS/MS and GC–MS/MS, respectively. No chemicals above the maximum residue levels were reported (data not shown).

### Plant-Based Bioassays Using Arabidopsis as the Model Plant

#### Root Development

Arabidopsis (Col-0) seeds were chlorine gas-sterilized for 3 h (Lindsey et al., 2017), sown on full strength Murashige and Skoog medium, and vernalized for 3 days at 4°C (Trinh et al., 2018). Next, the plates were exposed 8 h to light (room temperature 21.4°C, 40–60% humidity, light intensity 140 μmol m^–2^ s^–1^, 14/10 h day/night photoperiod) and transferred to the dark for another 3 days to induce etiolation. Then, uniform-size of 3 DAG (days after germination) seedlings were transplanted onto the growth medium and incubated vertically for 10 days. For the dose-response experiment, the medium was supplemented with 0.1, 0.5, 1, 2, 3, 3.67, 4, and 5 g/L SBE. For the time-course analysis of primary root development, the medium was supplemented with 1 g/L or 3 g/L SBE and incubated for 6, 12, and 24 h after etiolation. Seedlings were scored for the numbers of adventitious roots (ARs), junction roots (JRs), and lateral roots (LRs) under a binocular microscope (Olympus, SZX9, Tokyo, Japan). The primary root length (PRL) was measured by image analysis using ImageJ software version 1.53n (Schindelin et al., 2012) coupled with the “NeuronJ” plugin (Meijering et al., 2004). Each treatment consisted of three plates, and each plate contained 10 seedlings.

#### Shoot Growth

Arabidopsis shoot growth in response to SBE application was evaluated using a shoot assay adapted from De Diego et al. (2017). Briefly, seedlings were grown as described in the root assay and, at 3 DAG, transferred to 24-well tissue culture plates (VWR, CA, United States) containing Murashige and Skoog medium (4 mL per well) supplemented with 0.5 g/L SBE and 100 mM NaCl. Since at 100 mM NaCl, Arabidopsis rosettes are more compact (Claeys et al., 2014), we assessed the shoot growth at 13 DAG by measuring the green surface area using ImageJ. Each data point corresponds to 16 seedlings per treatment. The electrolyte leakage (also called conductivity) from seedlings was measured with a conductivity meter (inoLab Cond level 1) (Jiang et al., 2017).

#### Floating Leaf Disc

The senescence leaf disc assay was modified from Ghosh et al. (2015) and Chiu et al. (2021). Arabidopsis was cultivated in Jiffy-7^®^ peat pellets (Jiffy Products International AS, Norway) in a growth room (room temperature 18°C, less than 70% relative humidity, light intensity 100 μmol m^–2^ s^–1^, 16/8 h day/night photoperiod). The chlorophyll content in fully expanded rosette leaves of the same developmental stage was determined by the SPAD-502 chlorophyll meter (Konica Minolta, Tokyo, Japan). Leaves with SPAD values from 25 to 35 were harvested from healthy plants at 30 DAG. Around 20 leaf discs were punched with a 7 mm cork borer and floated on a 5 mL solution in each petri dish (55 mm diameter). Each treatment consisted of six individual plates. Distilled water (dH_2_O) was considered the blank, while 200 mM NaCl was considered the salt treatment. For exogenous SBE treatment, leaf discs were pretreated with 5 mL of dH_2_O or 0.5 g/L SBE for 1 day before incubating in the blank or salt solution for another 2 days. The plates were sealed with 3M Micropore tape (3M, St. Paul, MN, United States) and placed in the growth room (room temperature 25°C, 40–60% humidity, light intensity 200 μmol m^–2^ s^–1^, 24 h light photoperiod). After incubation, the leaf disc samples were first wrapped in dust-free tissue paper, then homogenized into powder with liquid nitrogen, and stored at −80°C for further analysis. Photosynthetic pigments of chlorophyll *a* (Chl *a*), chlorophyll *b* (Chl *b*), and carotenoids (Car) were assessed from frozen samples using a microplate reader (Tecan Infinite M200), according to Chiu et al. (2021). Malondialdehyde (MDA), as a biomarker of lipid peroxidation, was analyzed via the thiobarbituric acid (TBA)-reactive substances assay following (Hodges et al., 1999).

#### True Leaf Development

The true leaf assay was adapted from Rosa and Scheid (2014). Arabidopsis seeds were sown directly on the treated medium and vernalized as mentioned before (Trinh et al., 2018). Since the addition of NaCl (100 mM) in the medium induced moderate salt stress (Claeys et al., 2014), medium supplemented with 0.5 g/L SBE was prepared with or without 100 mM NaCl for the exogenous SBE application. The plates were positioned horizontally for germination in the light without etiolation and early leaf development monitoring for 10 days. The germination rate and the early development phenotypes were recorded daily at the same time each day. At 2 DAG, seed germination was evaluated by checking if the radicle was visible after testa rupture. Later, at 10 DAG, the successful emergence and expansion of the first pair of true leaves were determined by a binocular microscope at 20× zoom. If the side-view width of the true leaf had expanded larger than the hypocotyl diameter, it was then scored as a plant with expanded true leaves (Supplementary Figure 1). The percentage (%) of seedlings with true leaves was calculated following Equation 1:


(1) $$\%\mathrm{plants}\mathrm{with}\mathrm{true}\mathrm{leaves}=\frac{\mathrm{Number}\,\mathrm{of}\,\mathrm{seedlings}\,\mathrm{developed}\,\mathrm{with}\,\mathrm{true}\,\mathrm{leaves}}{\mathrm{Number}\,\mathrm{of}\,\mathrm{germinated}\,\mathrm{seedlings}}$$

This assay was performed as nine replicates for each treatment, and each plate contained 25 seeds. The harvested samples at 10 DAG were pooled into four biological replications and stored at −80°C for further analysis. The fresh weight of whole seedlings was measured and subsequently dried at 65°C for 48 h in an oven for dry biomass determination. In addition, the conductivity and MDA content in seedlings were measured as mentioned above to evaluate plasma membrane damage.

Hydrogen peroxide (H_2_O_2_), as one of the main ROS products, was quantified based on potassium iodide (KI) oxidation (Junglee et al., 2014). To detect *in situ* H_2_O_2_, histological staining with 3,3′-diaminobenzidine (DAB) on whole seedlings was performed as previously described (Daudi and O’Brien, 2012). The seedlings were imaged via an Olympus BX51 microscope (Olympus, Tokyo, Japan) equipped with differential interference contrast (DIC) optics at 10× zoom. The relative DAB staining intensity was calibrated in pseudo color and quantified in four tissues, inclusive of cotyledons, hypocotyls, shoot apical meristems (SAMs), and root, using ImageJ coped with the “Colour Deconvolution 2” plugin (Ruifrok and Johnston, 2001; Landini et al., 2021).

The antioxidant enzyme activity was then determined following a semi high-throughput protocol (Fimognari et al., 2020) with the adapted extraction method (Noctor et al., 2016). About 300 mg grounded sample was extracted with 2 mL extraction buffer (0.1 M phosphate buffer plus 1 M EDTA; pH 7.5) and 50 mg polyvinylpyrrolidone (PVP). The mixture was centrifuged for 10 min at 4°C. Next, the total protein content was quantified in the desalted supernatant by Wizard^®^ SV minicolumns using a spectrophotometer (DeNovix Inc., United States). Finally, the enzyme kinetic assays were performed for the activity measurement of ascorbate peroxidase (APX, EC:1.11.1.11), catalase (CAT, EC:1.11.1.6), glutathione reductase (GR, EC:1.8.1.7), glutathione *S*-transferase (GST, EC:2.5.1.18), monodehydroascorbate reductase (MR, EC:1.6.5.4), (cytoplasmic) peroxidase (POX, EC:1.11.1.5), and superoxide dismutase (SOD, EC:1.15.1.1). The antioxidative enzyme capacity, indicating the rate of catalyzed reaction by the enzyme, was calculated as a unit per mg of protein (Vanhoudt, 2014).

### Statistical Analysis

The treemap was generated indicating the chemical classification of compounds identified in SBE by R software version 4.1.1 (R Core Team, 2021) coupled with “treemap” package version 2.4-3 (Tennekes and Ellis, 2021). Non-parametric Kruskal–Wallis test was used with *post hoc* Dunn’s analysis (α = 0.05) for variances in root numbers of different types. PRL was normalized and fitted in a 5-parameter logistic model of dose-response analysis using “nplr” package version 0.1-7 (Commo and Bot, 2016). The half-maximal-effect concentration (EC_50_) was calculated on the PRL inhibition effect by getEstimates function (Venturelli et al., 2016). Dynamic growth models of plant leaf area were fitted in exponential growth curves illustrating the early development patterns. One-way ANOVA analysis was applied with *post hoc* Tukey HSD test (α = 0.05) to compare the treatment difference for other plant traits. Besides, two-way ANOVA analysis was used for two independent variables: the addition of growth media and timepoints. The statistical analysis of the remaining parameters and data visualization were performed with GraphPad Prism software version 8.0.2 (GraphPad, San Diego, CA, United States).

## Results

### Preparation of Sunflower Bark Extract and Chemical Analysis

Aqueous extraction of sunflower bark was obtained using a twin-screw extruder as described in the materials and methods. Supplementary Table 2 shows the chemical composition of the starting bark materials, with the insoluble fraction constituting 88% of the bark containing 50% cellulose and 15% lignin. The high content in lignocellulose promoted the separation into fiber (pulp) and a liquid filtrate, which contained the water-soluble compounds. The chemical composition and antioxidant activity of the freeze-dried liquid filtrate (SBE) contained 1.5% (w/w) soluble protein and 0.7% (w/w) carbohydrate (Table 1). The polyphenol content (TPC and TFC) per gram dry biomass of SBE was 6.46 mg AsA equivalents and 29.68 mg CHA equivalents with phenolic acids, and 9.70 μg QE equivalents and 15.93 μg rutin equivalents with flavonoids. TAC of SBE was represented as IC_50_ from *in vitro* antioxidant assays, which were 20.66 and 117.34 TE equivalents in the DPPH and ABTS assay, respectively.

**TABLE 1:** Contents of soluble protein, digestible carbohydrate, polyphenols, and *in vitro* antioxidant capacity in SBE.

Parameters		SBE
Soluble protein content (mg g DW^–1^ BSA equivalent)		14.91 ± 0.41
Digestible carbohydrate content (mg g DW^–1^ D-glucose equivalent)		6.91 ± 0.53
Total phenolic content (TPC) (mg g DW^–1^)	AsA equivalent	6.46 ± 0.79
	CHA equivalent	29.68 ± 0.81
Total flavonoid content (TFC) (μg g DW^–1^)	QE equivalent	9.70 ± 0.29
	Rutin equivalent	15.93 ± 2.01
Total antioxidant capacity (TAC) (mg g DW^–1^ TEAC)	IC_50_ DPPH assay	20.66 ± 0.67
	IC_50_ ABTS assay	117.34 ± 3.34

*Values are represented as mean ± SD (n = 3). SBE, sunflower bark extract; DW, dry weight; BSA, bovine serum albumin; AsA, ascorbic acid; CHA, chlorogenic acid; QE, quercetin; TEAC, trolox equivalent antioxidant capacity; DPPH, 2,2-diphenyl-1-picrylhydrazyl; ABTS, 2,2′-azino-bis (3-ethylbenzothiazoline-6-sulfonic acid).*

**FIGURE 1:**
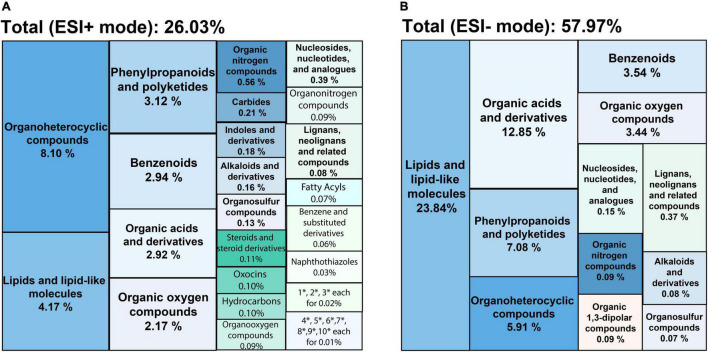
Treemaps of chemical classifications of SBE compounds detected by UHPLC-PDA-HRMS in **(A)** ESI+ and **(B)** ESI– modes. Each color shows the chemical taxonomy at the superclass level from ChemOnt ontology. Each value indicates the relative distribution of each superclass by total peak areas. SBE, sunflower bark extract; ESI+ (–), positive (negative) electrospray ionization. Where * is short for the superclasses following, 1: cinnamic acids and derivatives; 2: quinolines and derivatives; 3: imidazopyrimidines; 4: organic phosphoric acids and derivatives; 5: pyrroloazepines; 6: coumarins and derivatives; 7: pumiliotoxins, homopumiliotoxins, and allopumiliotoxins; 8: benzothiazoles; 9: azoles; 10: lupin alkaloids.

The low molecular weight primary and secondary metabolites were characterized by untargeted metabolic profiling using UHPLC-PDA-HRMS. It resulted in 2369 LC–MS features in the ESI+ mode and 814 under the ESI− mode (the chromatograms are shown in Supplementary Figure 2, all the identified compounds are listed in Supplementary Data 1). A total of 26.03% of the ESI+ and 57.97% of the ESI− detected peaks were tentatively identified. These compounds were classified according to 35 distinct categories of plant metabolites (Figure 1). The lipids and lipid-like molecules, organic acids, phenylpropanoids and polyketides, benzenoids, and organoheterocyclic compounds formed the five most extensive groups representing half of the classified metabolites. SBE was particularly rich in compounds across chemical superclasses of phenylpropanoids and polyketides, organic acids and derivatives, and benzenoids, of which polyphenols are well-known antioxidants with cytoprotective activity (Kiokias et al., 2020; Šamec et al., 2021). Thereby, we further focused on the diverse proportions of non-flavonoid and flavonoid compounds of interest under the phenylpropanoids and polyketides superclass in SBE. Eleven polyphenol classes involved 20 subclasses, and 45 tentatively identified compounds were illustrated in Table 2.

**TABLE 2:** Polyphenolic compounds of interest in SBE.

Class^1^	Subclass^1^	Parent level 1^1^	Predicted formula	Adduct	RT (min)	Precursor m/z	Area (×10^5^)	Total score^2^
2-Arylbenzofuran flavonoids	NA	2-Arylbenzofuran flavonoids	C_25_H_28_O_10_	[M + H]^+^	7.97	489.1735	0.14	5.90
Cinnamic acids and derivatives	Cinnamic acid esters	Cinnamic acid esters	C_11_H_12_O_2_	[M + H]^+^	7.75	177.0916	0.65	5.51
	Cinnamic acids	Cinnamic acids	C_9_H_8_O_2_	[M + H]^+^	23.46	149.0597	0.53	6.33
	Hydroxycinnamic acids and derivatives	Coumaric acids and derivatives	C_17_H_16_O_4_	[M + H]^+^	9.79	285.1128	0.12	5.78
		Hydroxycinnamic acids	C_9_H_8_O_3_	[M + H]^+^	4.96	165.0547	0.39	6.14
Coumarins and derivatives	Hydroxycoumarins	6,7-Dihydroxycoumarins	C_9_H_6_O_4_	[M + H]^+^	4.20	179.0345	0.35	5.78
		7-Hydroxycoumarins	C_10_H_8_O_4_	[M + H]^+^	4.80	193.0505	1.43	5.98
	Furanocoumarins	Angular furanocoumarins	C_17_H_12_O_6_	[M + H − H_2_O]^+^	6.04	295.0606	0.23	6.09
	Pyranocoumarins	Angular pyranocoumarins	C_21_H_22_O_7_	[M + H]^+^	7.89	387.1450	0.22	5.98
	Coumarin glycosides	Coumarin glycosides	C_15_H_16_O_9_	[M + H]^+^	4.20	341.0872	0.24	5.84
	NA	Coumarins and derivatives	C_9_H_6_O_2_	[M + H]^+^	6.44	147.0443	0.36	5.99
	Furanocoumarins	Linear furanocoumarins	C_13_H_10_O_5_	[M + H − H_2_O]^+^	7.34	229.0500	0.23	5.55
Flavonoids	Flavones	3′-Prenylated flavones	C_20_H_18_O_7_	[M + H − H_2_O]^+^	8.22	353.1040	0.15	5.75
	Flavans	8-Prenylated flavans	C_20_H_20_O_5_	[M + H]^+^	9.13	341.1380	0.22	6.32
	Flavonoid glycosides	Flavonoid *O*-glycosides	C_21_H_20_O_11_	[M + H]^+^	6.30	449.1083	0.11	6.43
	O-Methylated flavonoids	3-O-methylated flavonoids	C_17_H_12_O_8_	[M + H]^+^	7.20	345.0626	0.29	5.74
		8-O-methylated flavonoids	C_21_H_22_O_8_	[M + H]^+^	7.81	403.1376	1.58	6.04
Isoflavonoids	O-Methylated isoflavonoids	4′-O-methylated isoflavonoids	C_22_H_22_O_6_	[M + H]^+^	7.78	383.1476	0.17	5.97
	Isoflavans	Isoflavanols	C_15_H_14_O_3_	[M + H]^+^	6.90	243.1024	0.24	5.74
	Isoflavans	Isoflavanones	C_20_H_20_O_6_	[M + H]^+^	8.07	357.1351	0.93	6.28
	Isoflav-2-enes	Isoflavones	C_15_H_10_O_5_	[M + H]^+^	5.38	271.0605	0.18	6.30
	Isoflavonoid *O*-glycosides	Isoflavonoid *O*-glycosides	C_21_H_20_O_9_	[M + H]^+^	8.36	417.1198	0.33	6.52
	Furanoisoflavonoids	Pterocarpans	C_20_H_18_O_5_	[M + H − H_2_O]^+^	6.93	321.1141	0.19	6.37
Stilbenes	Stilbene glycosides	Stilbene glycosides	C_21_H_24_O_8_	[M + H]^+^	6.24	405.1557	0.21	6.07
Tannins	Hydrolyzable tannins	Hydrolyzable tannins	C_33_H_32_O_11_	[M + H]^+^	8.79	605.2021	0.21	5.38
2-Arylbenzofuran flavonoids	NA	2-Arylbenzofuran flavonoids	C_26_H_34_O_11_	[M − H]^–^	6.81	521.2031	0.38	5.63
Coumarins and derivatives	Furanocoumarins	Angular furanocoumarins	C_17_H_12_O_6_	[M − H]^–^	6.60	311.0547	0.56	5.95
	Coumarin glycosides	Coumarin glycosides	C_16_H_18_O_9_	[M − H]^–^	3.84	353.0870	2.45	6.00
	NA	Coumarins and derivatives	C_10_H_8_O_6_S	[M − H]^–^	7.06	254.9952	0.50	4.94
Cinnamic acids and derivatives	Hydroxycinnamic acids and derivatives	Coumaric acids and derivatives	C_13_H_16_O_5_	[M − H]^–^	8.12	251.0909	0.19	5.46
		Hydroxycinnamic acids	C_9_H_8_O_4_	[M − H]^–^	6.19	179.0340	25.60	6.24
Diarylheptanoids	Linear diarylheptanoids	Linear diarylheptanoids	C_20_H_24_O_3_	[M − H]^–^	13.86	311.1670	6.64	5.30
Flavonoids	Flavones	3-Prenylated flavones	C_25_H_24_O_7_	[M − H]^–^	7.81	435.1488	0.18	5.15
	O-Methylated flavonoids	3′-O-methylated flavonoids	C_16_H_12_O_6_	[M − H]^–^	6.48	299.0578	7.24	6.38
		4′-O-methylated flavonoids	C_16_H_12_O_5_	[M − H]^–^	8.45	283.0631	0.81	6.44
		6-O-methylated flavonoids	C_18_H_18_O_8_	[M − H]^–^	4.77	361.0936	0.23	5.52
		7-O-methylated flavonoids	C_20_H_20_O_8_	[M − H]^–^	8.22	387.1066	0.53	6.84
		8-O-methylated flavonoids	C_19_H_18_O_7_	[M − H]^–^	8.36	357.0961	0.37	5.92
	Flavonoid glycosides	Flavonoid *O*-glycosides	C_26_H_28_O_12_	[M − H]^–^	7.45	531.1503	0.53	6.47
	Flavones	Flavonols	C_16_H_12_O_7_	[M − H]^–^	7.39	315.0530	1.17	6.20
Isoflavonoids	Isoflavonoid *O*-glycosides	Isoflavonoid *O*-glycosides	C_24_H_24_O_11_	[M − H]^–^	5.86	487.1252	0.20	5.94
Isocoumarins and derivatives	NA	Isocoumarins and derivatives	C_13_H_14_O_7_	[M − H]^–^	8.11	281.0659	0.17	5.05
Linear 1,3-diarylpropanoids	Chalcones and dihydrochalcones	2′-Hydroxy-dihydrochalcones	C_21_H_24_O_5_	[M − H]^–^	18.83	355.1559	0.75	5.06
Phenylpropanoic acids	NA	Phenylpropanoic acids	C_9_H_10_O_5_	[M − H]^–^	3.33	197.0450	1.16	5.67
Tannins	Hydrolyzable tannins	Hydrolyzable tannins	C_14_H_18_O_9_	[M − H]^–^	6.91	329.0877	0.20	5.64

*The compounds were detected and tentatively identified via UHPLC-PDA-HRMS under ESI+ and ESI− modes.*

*SBE, sunflower bark extract; ESI+ (−), positive (negative) electrospray ionization; RT, retention time; NA, not available.*

*^1^The ontology information of identified features (class, subclass, and parent level 1) was retrieved by InChIKey according to ChemOnt chemical taxonomy (Djoumbou Feunang et al., 2016).*

*^2^The top-ranking score of the candidate compound was selected in the MS-FINDER program (Tsugawa et al., 2016).*

### Sunflower Bark Extract Inhibited the Primary Root Growth of Non-stressed Arabidopsis Seedlings in a Dose-Response Manner

**FIGURE 2:**
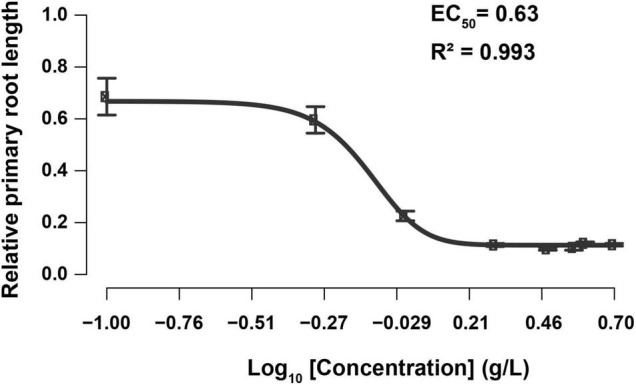
Dose-response curve of relative primary root length to the increase of SBE concentration in the Arabidopsis root assay. Each data point indicates the mean ± SD (*n* > 15). The black line represents the 5-parameter logistic regression. SBE, sunflower bark extract; EC_50_, half-maximal-effect concentration.

The root tip is a sensory organ that evaluates the presence of mineral nutrients and physical obstructions, adapting its growth in response to the conditions by altering the growth rate and orientation of cell division and expansion (Svolacchia et al., 2020). Hence, the primary root is very susceptible to environmental conditions, and its plasticity facilitates the detection of slight changes in the composition of the growing medium (Malamy, 2005). To test the impact of SBE application on root growth, root length (PRL) and branching (AR, LR, and JR) of *in vitro* grown Arabidopsis seedlings were determined (Supplementary Figure 3). Significantly less AR was formed when treated with SBE at higher doses. A strong reduction in LR number was observed at SBE levels from 1 g/L, while the effect on JR formation was more complex with a promotion up to 1 g/L SBE but a reduction from a higher concentration above 1 g/L. Furthermore, the EC_50_ of SBE inhibition of PRL was 0.63 g/L (Figure 2). Since root growth often shows an adaptive behavior to exogenous stimuli, the inhibition of PRL was examined at different time intervals after transfer to the medium containing SBE. Growth inhibition was observed 24 h after treatment with 1 g/L SBE, while at 3 g/L SBE inhibition occurred 6 h after treatment (Supplementary Figure 4). To avoid secondary effects following primary root growth inhibition, SBE was applied at 0.5 g/L in the subsequent assays (Supplementary Figure 3).

### Priming With Sunflower Bark Extract Did Not Alter Germination Rate but Stimulated Shoot Growth

**FIGURE 3:**
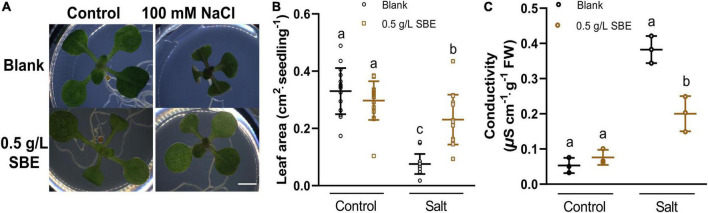
Salt stress alleviation of SBE treatment in the Arabidopsis shoot assay. **(A)** Phenotypes of representative seedlings at 13 DAG grown on multiwell plates. Bar = 2 mm. The changes of **(B)** leaf area (*n* = 16) and **(C)** conductivity (*n* = 4) of 13 DAG seedlings. Error bars indicate SDs of the means. Different letters represent significant differences between treatments using Tukey’s HSD test (*p* < 0.05). SBE, sunflower bark extract; DAG, days after germination.

**FIGURE 4:**
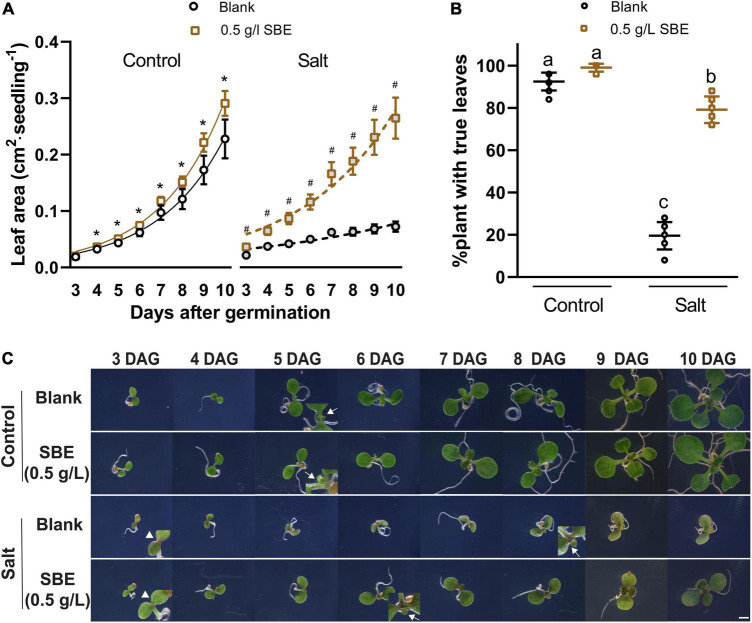
Salt stress alleviation of SBE treatment in the Arabidopsis true leaf assay. **(A)** The dynamic growth of leaf area from 3 DAG to 10 DAG. Error bars indicate SDs of the means (*n* = 9). Different letters represent significant differences between treatments using Tukey’s HSD test (*p* < 0.05). Fitted lines were exponential curves of dynamic leaf area growth. The symbols * and ^#^ represent significant differences between treatment with or without SBE addition under control or salt stress conditions at the same time point, respectively (*p* < 0.05). Both salt treatments were significantly different from the two non-stress treatments at every time point (label not shown). **(B)** The percentages of plants successfully developed with true leaves at 10 DAG. **(C)** Phenotype changes of representative seedlings from 3 DAG to 10 DAG focusing on the center of two cotyledons under binocular 6.3× zoom. Bar = 2 mm. Arrows without tails indicate salt stress alleviation by SBE addition on anthocyanin accumulation at the cotyledon edges of 3 DAG seedlings. Arrows with tails showed the earliest starting time point of visible true leaves. SBE, sunflower bark extract; DAG, days after germination.

We did not observe a notable change in shoot growth after transferring 3 DAG Arabidopsis seedlings to SBE containing medium (Figure 3B). In addition to the seed priming experiment, there was no impairment of seed germination rate after 2 days in any of the treatments (Supplementary Figure 5). After 4 days, however, we observed a slight, statistically significant increase in the leaf area of seedlings grown on SBE containing medium (Figure 4A).

### Sunflower Bark Extract Alleviated Shoot Growth Inhibition Under Salt Stress

Biostimulants typically show a more pronounced effect in plants grown under stress (Rouphael and Colla, 2020). The impact of SBE was therefore assessed under conditions of salt stress. As the suppression of shoot growth by NaCl was very notable (Claeys et al., 2014), the projected leaf area was used as a proxy for determining the effect of SBE. In the shoot assay, the shoot growth was severely inhibited in the presence of 100 mM NaCl, showing a reduced petiole length, smaller cotyledons, and delayed or even arrested emergence of the first true leaves (Figure 3A). In the presence of SBE, the NaCl-stressed seedlings generated a significantly larger green surface area than in control salt-stressed plants (Figure 3B). NaCl causes osmotic stress and ionic imbalances, affecting the integrity of the plasma membrane (Dubois and Inzé, 2020). Therefore, the seedlings were collected after 13 DAG to determine the electrolyte leakage (Figure 3C). Salt stressed plants grown on SBE containing medium showed much lower conductivity than the control plants, suggesting that SBE treatment protected the plants from cell membrane damage.

We then put more attention to early true leaf development in seed priming treatment. SBE did not influence the germination rate of Arabidopsis under salt stress conditions (Supplementary Figure 5). Since we noticed that shoot growth was enhanced in SBE containing medium, a time-course analysis was performed to determine this response in more detail (Figure 4). On 100 mM NaCl-containing medium, shoot development was significantly reduced, while this growth inhibition was strongly alleviated when SBE was included in the medium (Figure 4A). Under normal conditions, all plants expanded their first true leaves at 10 DAG, while in the presence of NaCl, only 20% of the plants produced expanded true leaves. However, the number of salt-stressed plants with expanded true leaves increased to around 80% when treated with SBE (Figure 4B). The addition of SBE advanced true leaf development by about 2 days, and the effects were already noticeable from 3 DAG when NaCl-induced anthocyanin accumulation as a red discoloring of the cotyledons and at the upper hypocotyl margin was observed. While less intense red coloring was shown in the presence of SBE (Figure 4C).

### Sunflower Bark Extract Preserved Photosynthesis Pigments and Stabilized the Cell Membrane Under Salt Stress

**FIGURE 5:**
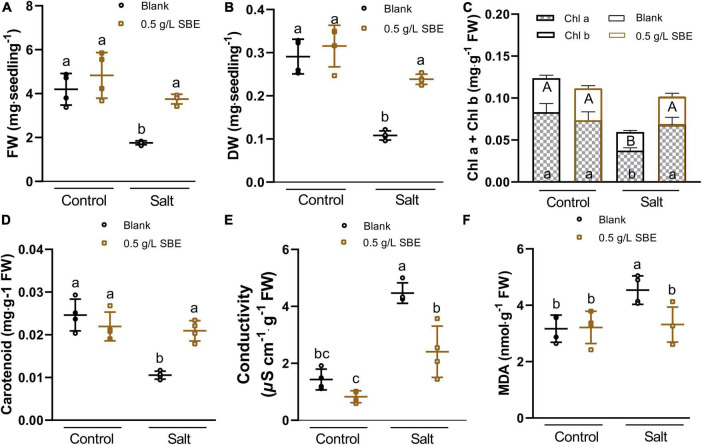
Salt stress alleviation of SBE treatment on the physiological traits of 10 DAG Arabidopsis in the true leaf assay. **(A)** Fresh weight, and **(B)** dry weight of the whole seedlings at 10 DAG. The changes of photosynthetic pigments containing **(C)** chlorophyll a and b, **(D)** carotenoid content, **(E)** conductivity, and **(F)** MDA content. Error bars indicate SDs of the means (*n* = 4). Different letters represent significant differences between treatments using Tukey’s HSD test (*p* < 0.05). SBE, sunflower bark extract; DAG, days after germination; FW, fresh weight; DW, dry weight; Chl a, chlorophyll a; Chl b, chlorophyll b; MDA, malondialdehyde.

The Arabidopsis response to salt stress includes a reduction in growth, reflected in lower fresh weight and dry weight (Figures 5A,B), and a bleaching effect that entails a decline of pigments, the photosynthetic chlorophyll, and carotenoids (Leschevin et al., 2021). These pigments were quantified, and chl*_*a*_*_+_*_*b*_* was reduced twofold, whereas carotenoids were down by about threefold in 10 DAG salt-stressed seedlings (Figures 5C,D). This protective effect of SBE was accompanied by diminished salt stress-induced electrolyte leakage (Figure 5E) and MDA overaccumulation (Figure 5F).

**FIGURE 6:**
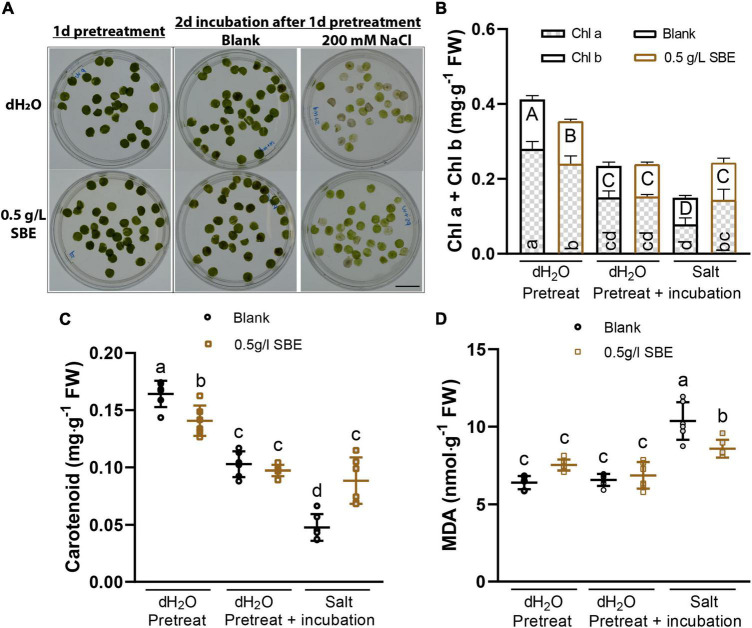
Salt stress alleviation of SBE pretreatment on expanded Arabidopsis leaves in the leaf disc assay. **(A)** The representative image of treated leaf discs was taken before sampling. Bar = 2 cm. Photosynthetic pigments contain **(B)** chlorophyll *a* and *b*, and **(C)** carotenoid content. **(D)** MDA content. Error bars indicate SDs of the means (*n* = 6). Different letters represent significant differences between treatments using Tukey’s HSD test (*p* < 0.05). SBE, sunflower bark extract; DAG, days after germination; FW, fresh weight; Chl a, chlorophyll a; Chl b, chlorophyll b; MDA, malondialdehyde.

To investigate whether SBE exerts a priming protective effect on mature leaves, punched leaf discs from fully developed leaves were pretreated for 1 day with SBE or water as a control (Figure 6A). Next, the leaves were floated for 2 days on a solution with or without 200 mM NaCl. The SBE pretreated leaves maintained a higher level of chl*_*a*_*_+_*_*b*_* and carotenoid content under salt stress conditions (Figures 6B,C). Similar to the salt-stressed seedlings, SBE pretreatment dampened the accumulation of MDA production induced by salt (Figure 6D).

### Sunflower Bark Extract Mitigates NaCl Toxicity by Suppressing Hydrogen Peroxide Overaccumulation

**FIGURE 7:**
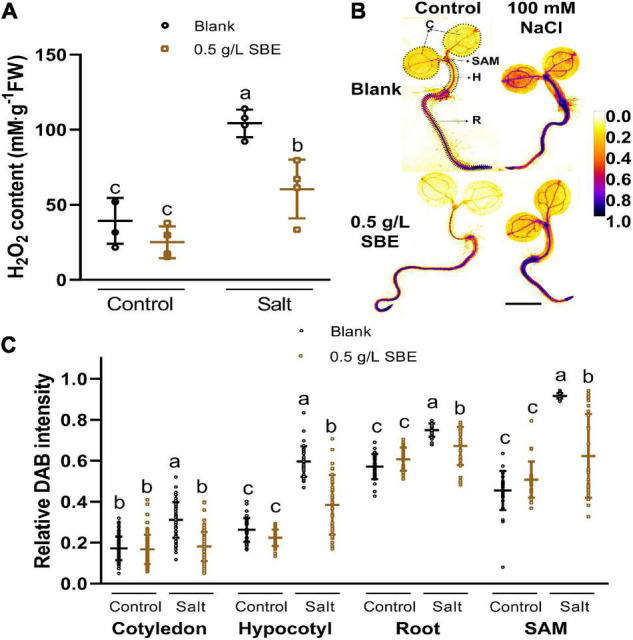
Hydrogen peroxide accumulation of 3 DAG and 10 DAG Arabidopsis seedlings under salt stress in the true leaf assay. **(A)** H_2_O_2_ content in 10 DAG seedlings. **(B)** Representative images of 3 DAG seedlings after histological staining of H_2_O_2_ by DAB are shown in pseudo-colors of relative DAB intensity as shown in the color bar. Regions of interested organs are shown in circles with black dotted lines. Bar = 1 mm. **(C)** Relative DAB staining intensity of four organs in 3 DAG seedlings. Error bars indicate SDs of the means [*n* = 4 **(A)** and >25 **(B)**]. Different letters represent significant differences between treatments (*p* < 0.05). SBE, sunflower bark extract; DAG, days after germination; H_2_O_2_, hydrogen peroxide; DAB, 3′,3-diaminobenzidine; C, cotyledon; H, hypocotyl; R, root; SAM, shoot apical meristem.

Salinity-induced osmotic and ionic stress affects cellular redox homeostasis by H_2_O_2_ overproduction causing oxidative damage to proteins and lipids (Munns and Tester, 2008; Huang et al., 2019). We, therefore, asked if SBE scavenges ROS in salt-stressed plants. The H_2_O_2_ levels more than doubled in salt stress seedlings at 10 DAG (Figure 7A). The salt-induced increase in H_2_O_2_ was reduced to about 60% by SBE application (Figure 7A). ROS reduction was already apparent after 3 DAG upon ROS staining with DAB (Supplementary Figure 6 and Figure 7B), which coincides with the earliest time point when SBE started showing a significant improvement in shoot growth on NaCl containing medium (Figure 4C). The relative DAB staining intensity in plants grown in the presence of SBE was lower than in plants without SBE (Figure 7B). The quantification of DAB intensity in the cotyledons, hypocotyl, root, and SAM showed that the dampening effect of SBE occurred in all seedling organs (Figure 7C). H_2_O_2_ levels were relatively higher in the root and SAM than in cotyledons and hypocotyl. The SBE mediated reduction of ROS was most pronounced in the SAM in line with the protective effect of SBE on true leaf development under salt stress conditions.

**FIGURE 8:**
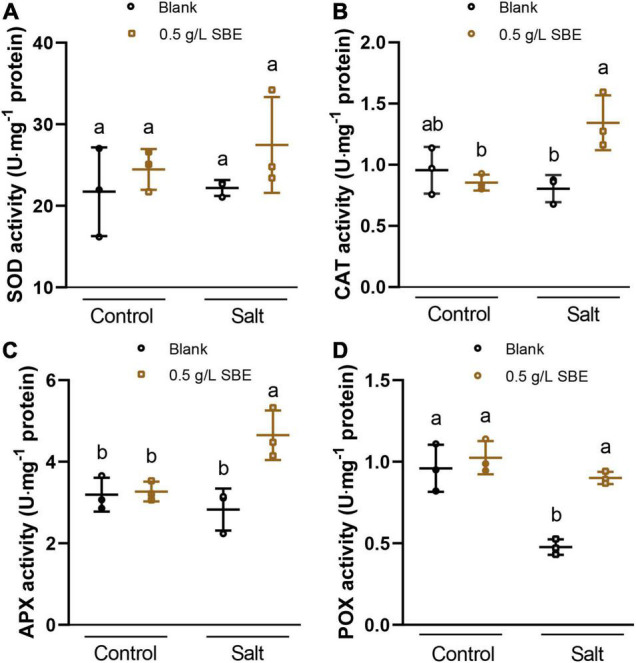
The antioxidant activities of Arabidopsis seedlings under salt stress at 10 DAG in the true leaf assay. **(A)** SOD activity, **(B)** CAT activity, **(C)** APX activity, **(D)** POX activity. Error bars indicate SDs of the means. Different letters represent significant differences between treatments using Tukey’s HSD test (*p* < 0.05). SBE, sunflower bark extract; DAG, days after germination; U, enzyme activity unit (μmol/min); SOD, superoxide dismutase; CAT, catalase; APX, ascorbate peroxidase; POX, peroxidases.

Next, we asked whether SBE neutralizes ROS formation via the overactivation of antioxidant enzymes that are part of the plant defense system (Apel and Hirt, 2004; Bobrovskikh et al., 2020). To this end, the activity of the antioxidant enzymes was measured in control and salt-stressed seedlings grown with or without SBE supplement (Figure 8). In control conditions, no difference in antioxidant enzymes activity was investigated. SBE treatment of salt-stressed plants significantly increased the activity of CAT, APX, and POX (Figures 8B–D), which directly participate in eliminating ROS pathway by catalyzing the conversion from H_2_O_2_ to H_2_O (Dumanović et al., 2021). In contrast, SOD, GR, GST, and MR were not significantly altered by SBE treatment (Figure 8A and Supplementary Figure 7).

## Discussion

This study on the analysis of potential biostimulant activity is a major step in valorizing sunflower stalks. Here, we report that SBE, a side stream obtained during fiber isolation by twin-screw extrusion, contains polyphenols and other bioactive molecules that activate ROS scavenging enzymes, thereby suppressing the oxidative damage to Arabidopsis seedlings grown on the NaCl-containing medium. Together, the results suggest that SBE is a potential source of biostimulant.

The twin-screw extrusion method is an extraction technique for separating insoluble parts (e.g., fibers) from solvent-soluble molecules in a single step. The solvent, thermal and mechanical actions are customized to extract fiber-rich plant biomass from crop waste such as sunflower stems (Evon et al., 2018; Vandenbossche et al., 2019). The extrusion method is an ecofriendly biorefinery process that meets the criteria of “green chemistry” extraction of natural products (Chemat et al., 2012). Yet, the bark extract is not economically valorized from the sunflower fiber part. SBE is an aqueous extract adhering to the guidelines of the European directive for certification of natural substances without pesticide residue and, as such, can be used to produce a novel biostimulant.

The outer bark constitutes about 90% dry weight of the whole stalk, indicating that it is much denser than the pith (Xu et al., 2020). The stems contain a considerable amount of polyphenols (Kamal, 2013). The chemical composition of an aqueous extract from sunflower bark has not yet been reported. An estimation of phenolics content (TPC) in SBE (Table 1) was obtained by applying a modified Folin–Ciocalteu assay to quantify TPC values against CHA (Sánchez-Rangel et al., 2013). However, the TFC values for evaluating “total” flavonoid content are not adequate based on aluminum complex reaction as the two procedures we performed are specific for different flavonoid structures (Pękal and Pyrzynska, 2014). In this situation, it is not possible to compare the accurate polyphenol content across the studies using different protocols. Therefore, to improve the coverage of present plant metabolites in SBE besides polyphenols, UHPLC-PDA-HRMS was performed for more precise detection and identification (Lai et al., 2018). Thanks to the validated algorithm increasing the accuracy of compound identification (Tsugawa et al., 2016), MS-DIAL combined with MS-FINDER is recommended to match MS^e^ spectra *in silico* for untargeted metabolomics (Blaženović et al., 2017; Vaniya et al., 2017). Our study thus provides the chemical composition of SBE, to some degree, revealing the plant metabolomics with both colorimetric and UHPLC-PDA-HRMS analysis.

Sunflower bark extract shows conspicuously *in vitro* activity, completely inhibiting primary root growth at doses above 1 g/L (Supplementary Figure 3). This strong growth inhibition contrasts with the growth-promoting effects observed in salt-stressed plants treated with more diluted SBE. Sunflowers produce a diverse set of allelochemicals that either positively or negatively affect the growth of other plant species (Macías et al., 2002). Some of these allelochemicals were already identified in sunflower stalks (Maheswari et al., 2019). Allelopathic activity in sunflower was closely linked with the presence of polyphenols and terpenes (Rawat et al., 2017). Sunflower aqueous shoot extract partially inhibited rapeseed and *Cephalaria* seedling growth (Hamad, 2017). Natural polyphenols were extensively reported to induce cytotoxicity in plant normal cells as well as cancer cell in dose-dependent manners (Rasouli et al., 2016; Perveen, 2017). These or other allelopathic compounds are likely also present in SBE and could be responsible for the primary root growth inhibition at high doses and may also prime to trigger a plant defense response, a property of many biostimulants (Kerchev et al., 2020).

However, at 0.5 g/L, SBE protected Arabidopsis from oxidative damage induced by a moderate concentration of NaCl in the medium (Figure 4). Also, allelopathic extracts from *Levisticum officinale* Koch were recently identified to have a positive performance on soybean yield (Szparaga et al., 2021). One of the reasons is that some polyphenols have antioxidant activity and play a role in controlling oxidative stress in plants (reviewed in Ferdinando et al., 2012; Šamec et al., 2021). For example, quercetin suppressed the ROS toxicity of paraquat in seedlings of Arabidopsis, tobacco, and duckweed (Kurepa et al., 2016) and heavy-metal stress in Arabidopsis (Zhang et al., 2017). Recent evidence shows that flavonoids are active as cytoprotective antioxidants preventing mitochondrial signaling that regulates autophagy and apoptosis (Kicinska and Jarmuszkiewicz, 2020). Phenolic acids are also stress-relieving molecules due to their high antioxidative properties (Šamec et al., 2021), namely, caffeic and sinapic acids enhance salt tolerance in wheat seedlings when exogenously applied (Kaur et al., 2017). The reduction in ROS levels mediated by SBE is likely due to the antioxidant activity of polyphenols and possibly other molecules within a non-toxic concentration range.

Next to polyphenols, SBE contained digestible carbohydrates and soluble protein. Sugars released from carbohydrates function as energy metabolites, osmoprotectants, and signaling molecules and mitigate stress responses in plants (Rook et al., 2006; Krasensky and Jonak, 2012). Likewise, several amino acids derived from proteins have been proved to be precursors of secondary metabolites and signaling molecules tightly related to plant responses under stress (Batista-Silva et al., 2019). These water-soluble primary compounds may support plant stress adaptation and complement the ROS suppressing activity of the above-mentioned bioactive agents.

The true leaf development assay in Arabidopsis is a sensitive method for evaluating DNA damaging agents (Rosa et al., 2013). High concentrations of NaCl induce ROS formation in germinating eggplant leading to DNA damage (Kiran et al., 2020). In particular stem cells in germinating seeds and in shoot meristems are highly susceptible to DNA damage causing an arrest in leaf development (Fulcher and Sablowski, 2009). We speculate that the accumulation of H_2_O_2_ in SAM causes oxidative damage, including DNA damage and that this prevents the development of the first true leaves in our experiments (Figure 7C). Polyphenols suppress ROS overaccumulation by neutralizing free radicals with donated electrons or hydrogen atoms with concomitant formation of stabilized phenolic radicals (Dumanović et al., 2021). In addition, polyphenols activate ROS scavenging enzymes (Kerchev et al., 2020). These enzymes function in plant defense and regulate cell growth and cell death (Mhamdi and Van Breusegem, 2018). Therefore, SBE will likely affect shoot growth under salt stress by activating ROS scavenging enzymes.

In future experiments, fractionation of SBE into less complex mixtures will be necessary to define the extent of synergism between the different bioactive molecules. Plant extract-based biostimulants are typically mixtures of bioactive compounds (García-García et al., 2020), which may explain why certain extracts are active despite the applied low dilutions. Given that the large biomass of sunflower stalks is currently underused, we anticipate that it is a suitable resource for biostimulant development and will contribute to valorization of the stems. Further studies will also focus on the consistency and reproducibility of bioactivity across separate harvested materials.

## Conclusion

Taken together, we demonstrated that SBE can be refined from sunflower bark using water as an extraction solvent in a twin-screw extruder and that it contains bioactive molecules that act as protectants against salt stress by maintaining cellular redox homeostasis. The results highlight the potential of SBE as a source for biostimulant production that can be used for seed biopriming, soil, and foliar application. Future studies are underway to test the effectiveness of SBE biostimulant under field conditions on various crops. The characterization of the bioactive ingredients is a critical target to unravel the chemical structure and underlying mode of action.

## Data Availability Statement

The raw data supporting the conclusions of this article will be made available by the authors, without undue reservation.

## Author Contributions

JL, AR, and DG: conceptualization. JL: methodology and investigation and writing – original draft preparation. HKT: initial screening. SB and PE: resources. PE and JL: chemical characterization. CVP, PM, SM, and JL: chemical profiling. PE, SB, CVP, SM, TVG, and DG: writing – review and editing. DG: supervision. TVG, AR, LX, and DG: project administration. BVD, SB, PE, SM, and DG: funding acquisition. All authors contributed to the article and approved the submitted version.

## Conflict of Interest

The authors declare that the research was conducted in the absence of any commercial or financial relationships that could be construed as a potential conflict of interest.

## Publisher’s Note

All claims expressed in this article are solely those of the authors and do not necessarily represent those of their affiliated organizations, or those of the publisher, the editors and the reviewers. Any product that may be evaluated in this article, or claim that may be made by its manufacturer, is not guaranteed or endorsed by the publisher.
