# Supplementary material for: Sunflower Bark Extract as a Biostimulant Suppresses Reactive Oxygen Species in Salt-Stressed Arabidopsis
Source: Front Plant Sci. 2022 Jul 1;13:837441. doi: 10.3389/fpls.2022.837441 (PMC9285015; doi:10.3389/fpls.2022.837441)
Supplement: Supplementary file 1 [file Data_Sheet_1.docx]

Supplementary Material

# Supplementary Data

**Supplementary Data 1.** All identified compounds in SBE by UHPLC-PDA-HRMS detection.

# Supplementary Figures and Tables

## Supplementary Figures

**Supplementary Figure 1.** Representative pictures of the failure (**A**) and the success (**B**) of true leaves expansion of 10 DAG Arabidopsis seedlings under salt stress were imaged with a binocular 20X zoom. We defined the seedlings with developed true leaves as those whose true leaf width was wider than that of the hypocotyl. White lines indicate the width of emerging true leaves and hypocotyls. Bar = 2 mm. DAG: days after germination.

**Supplementary Figure 2.** Representative chromatogram of SBE at a concentration of 1 mg/mL in water using UPLC-PDA-HRMS analysis under **(A)** ESI+ and **(B)** ESI- modes. SBE: sunflower bark extract; ESI+ (-): positive (negative) electrospray ionization.

**Supplementary Figure 3.** The dose-response effect of SBE treatment on phenotypic root traits of 13 DAG Arabidopsis seedlings in the root assay, including the number of AR **(A)**, JR **(B)**, LR **(C)**, and PRL **(D)**. Error bars represent the standard deviation of means. Different letters indicate significant differences between doses using Dunn’s test (*p* < 0.05). SBE: sunflower bark extract; DAG: days after germination; AR: Adventitious root; JR: junction root; LR: lateral root; PRL: primary root length.

**Supplementary Figure 4.** The primary root length at four different time points of 3 DAG Arabidopsis etiolated seedling transferred to media with or without SBE. Error bars represent the standard deviation of means. Stars indicate significant differences between treatments at the same time point using Tukey’s HSD test (*p* < 0.05). The variance within the same treatment between different time points was significant (label not shown). SBE: sunflower bark extract; DAG: days after germination.

**Supplementary Figure 5.** The percentage of seeds germinated at 2 DAG in the Arabidopsis true leaf assay. Error bars represent the standard deviation of means (n = 4). The same letters indicate a nonsignificant difference between doses using Tukey’s HSD test (*p* > 0.05). SBE: sunflower bark extract; DAG: days after germination.

**Supplementary Figure 6.** Representative pictures of 3 DAG Arabidopsis seedlings after histological staining of H_2_O_2_ by DAB in the true leaf assay. Bar = 1 mm. SBE: sunflower bark extract; DAG: days after germination; H_2_O_2_: hydrogen peroxide; DAB: 3’,3-diaminobenzidine.

**Supplementary Figure 7.** The activities of antioxidant enzymes in extracted Arabidopsis seedlings under salt stress at 10 DAG in the true leaf assay. **(A)** GST activity, **(B)** GR activity, **(C)** MR activity. Error bars indicate standard deviations of the means. The same letters represent significant differences between treatments using Tukey’s HSD test (*p* > 0.05). SBE: sunflower bark extract; DAG: days after germination; U: enzyme activity unit (μmol/min); GST: glutathione S-transferase; GR: glutathione reductase; MR: monodehydroascorbate reductase.

## Supplementary Tables.

**Supplementary Table 1.** MS-DIAL settings used for UHPLC-PDA-HRMS data processing.

| **Settings** | **Sub settings** | **Value** |  |
| --- | --- | --- | --- |
| **Project set-up** |  |  |  |
|  | MS1 Data type | Centroid |  |
|  | MS2 Data type | Centroid |  |
|  | Target | Metabolomics |  |
|  | Mode | diMSMS |  |
|  |  |  |  |
| **Data Collection** |  |  |  |
| Data Collection Parameters | Retention time begin | 1 min |  |
|  | Retention time end | 27 min |  |
|  | Mass Range Begin | 50 Da |  |
|  | Mass Range End | 1200 Da |  |
|  | MS2 mass range begin | 50 Da |  |
|  | MS2 mass range end | 1200 Da |  |
| **Centroid Parameters** | MS1 Tolerance | 0.05 Da |  |
|  | MS2 Tolerance | 0.1 Da |  |
| Isotope Recognition | Maximum Charged Number | 2 |  |
| Peak Detection |  |  |  |
| **Peak Detection Parameters** | Smoothing Method | Linear Weighted Moving Average |  |
|  | Smoothing Level | 3 |  |
|  | Minimum Peak Width | 5 scans |  |
|  | Minimum Peak Height | 3000 Amplitude in positive ion mode, 5000 Amplitude in negative ion mode |  |
| Peak Spotting Parameters | Mass Slice Width | 0.1 Da |  |
| Exclusion Mass List | mass | 556.2771 in positive ion mode, 554.2615 in negative ion mode |  |
|  | tolerance | 0.01 Da |  |
| MS2Dec |  |  |  |
| Deconvolution Parameters | Sigma Window Value | 0.5 |  |
| Purification Parameters | MS/MS abundance Cut Off | 0 Amplitude |  |
|  | Exclude After Precursor Ion | Check |  |
|  | Keep the Isotopic Ions Until | 0.5 Da |  |
|  | Keep the isotopic ions w/o MS2Dec | Uncheck |  |
| **Identification** |  |  |  |
| **MSP file and MS/MS Identification setting** | MSP File | MSMS-Public-Pos-VS15.msp for positive ion mode, MSMS-Public-Neg-VS15.mspfor negative ion mode |  |
|  | Retention Time Tolerance | 100 min |  |
|  | Accurate Mass Tolerance (MS1) | 0.01 Da |  |
|  | Accurate Mass Tolerance (MS2) | 0.05 Da |  |
|  | Identification Score Cut Off | 80% |  |
|  | **Use Retention Information For Scoring** | Uncheck |  |
| **Text File and Post Identification Setting** | **Uncheck** |  |  |
| Adduct | Included | [M+H]+, [M + Na]+, [M + H-H2O]+ , [M + ACN + H]+ in positive ion mode; [M-H]-, [M-H2O-H]-, in negtive ion mode |  |
| Alignment |  |  |  |
| Alignment Parameters Setting | Reference File | Random sample |  |
|  | Retention Time Tolerance | 0.1 min |  |
|  | MS1 Tolerance | 0.025 Da |  |
|  | Retention Time Factor | 0.5 |  |
|  | MS1 Factor | 0.5 |  |
|  | Peak Count Filter | 100% |  |
|  | N% Detected in at Least One Group | 100% |  |
| Isotope Tracking |  |  |  |
| Tracking of Isotope Labels | Uncheck |  |  |
| Ion mobility | Uncheck |  |  |

**Supplementary Table 2.** Chemical characterization of the dried sunflower bark used as starting material in the twin-screw extruder.

| **Parameters** | **Sunflower bark** |
| --- | --- |
| Minerals (% DW) | 3.4 ± 0.0 |
| Lipids (% DW) | 0.6 ± 0.1 |
| Cellulose (% DW) | 49.6 ± 0.6 |
| Hemicelluloses (% DW) | 23.1 ± 0.1 |
| Lignin (% DW) | 15.3 ± 0.7 |
| Water-soluble compounds (% DW) | 7.4 ± 0.1 |

Values represented as mean ± standard deviation. DW: Dry weight.
