# Supplementary figures and images for: Sunflower Bark Extract as a Biostimulant Suppresses Reactive Oxygen Species in Salt-Stressed Arabidopsis
Source: Front Plant Sci. 2022 Jul 1;13:837441. doi: 10.3389/fpls.2022.837441 (PMC9285015; doi:10.3389/fpls.2022.837441)

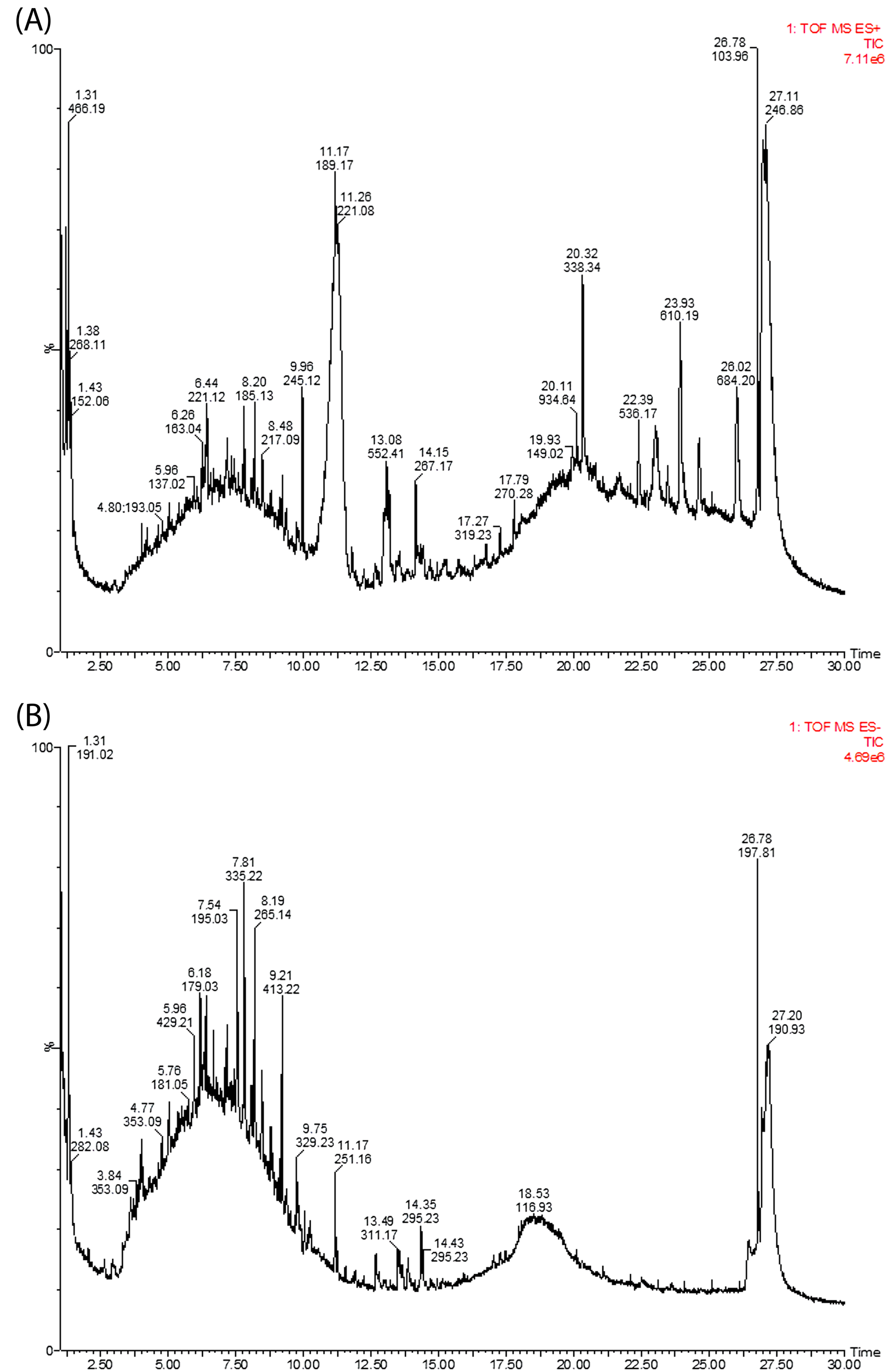

Supplement: Supplementary file 4 [file Image_2.TIF]

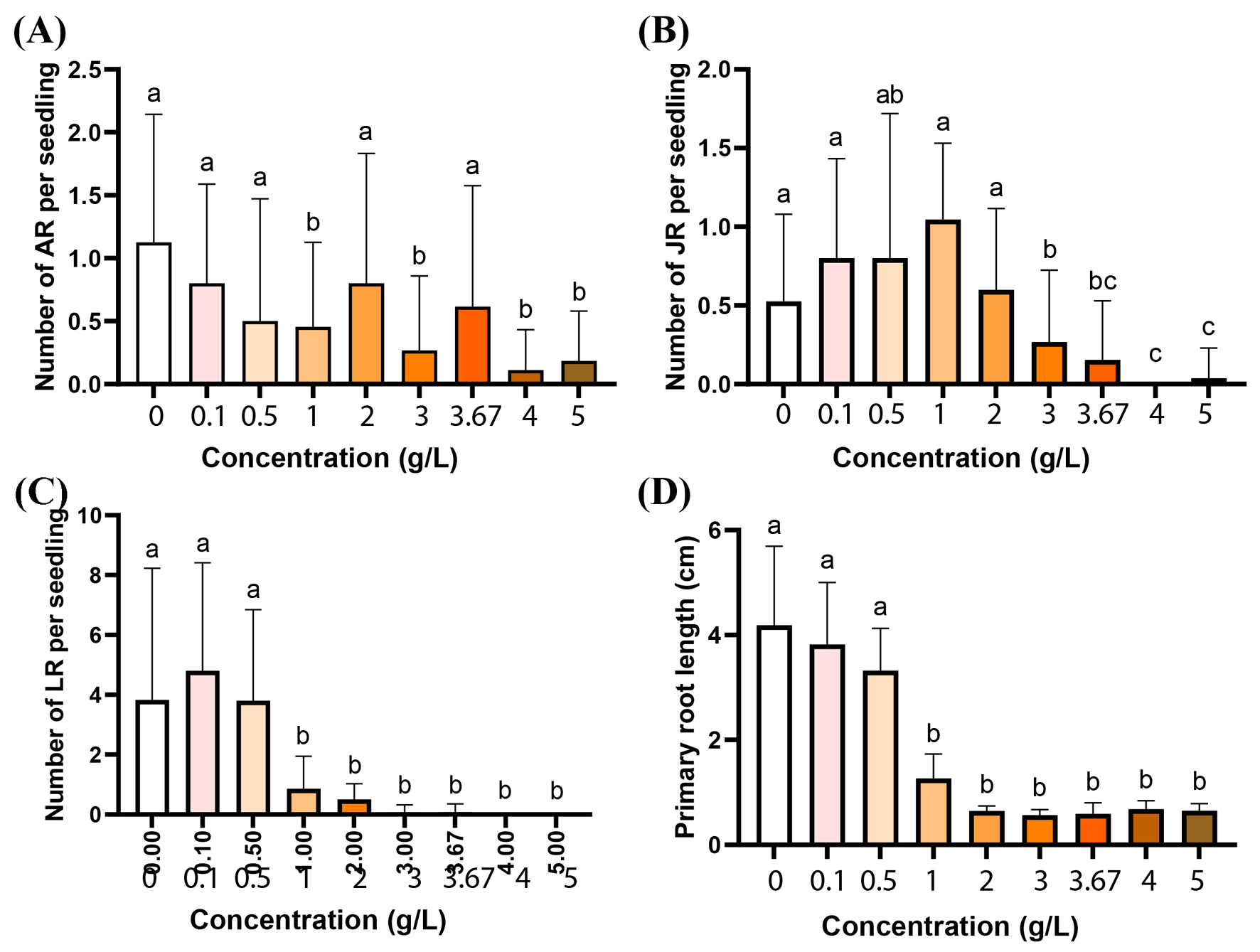

Supplement: Supplementary file 5 [file Image_3.TIF]

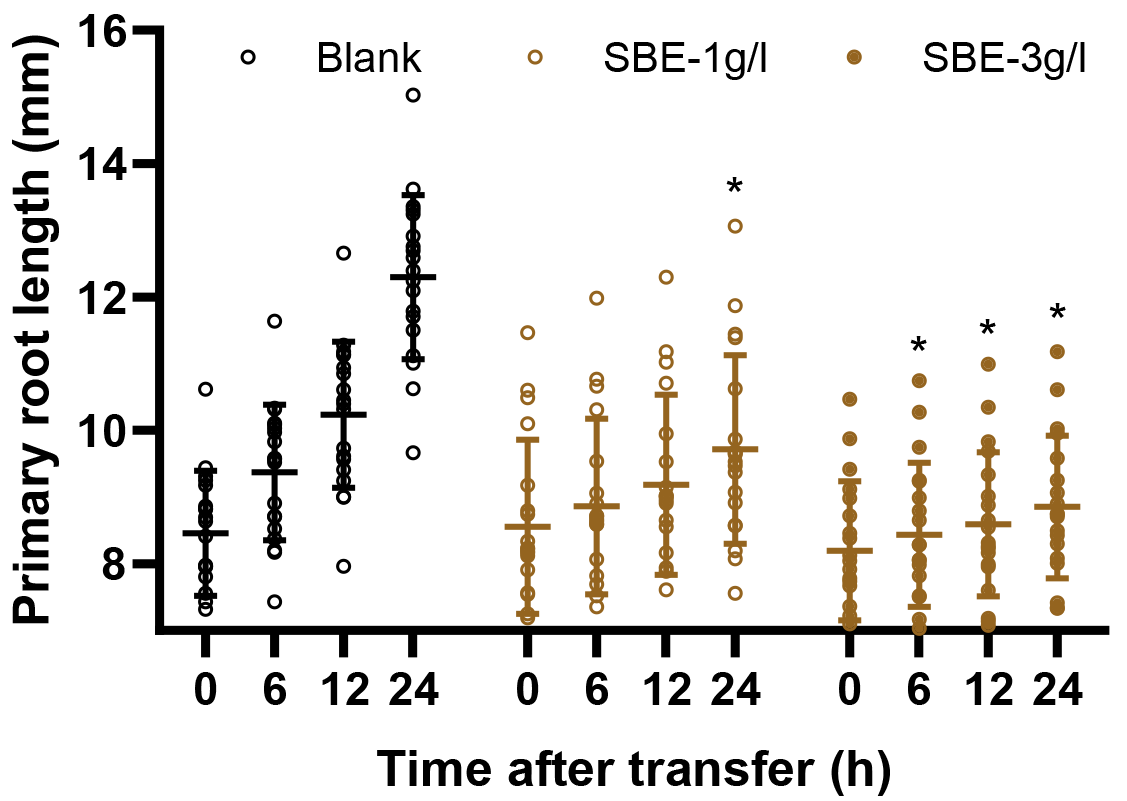

Supplement: Supplementary file 6 [file Image_4.TIF]

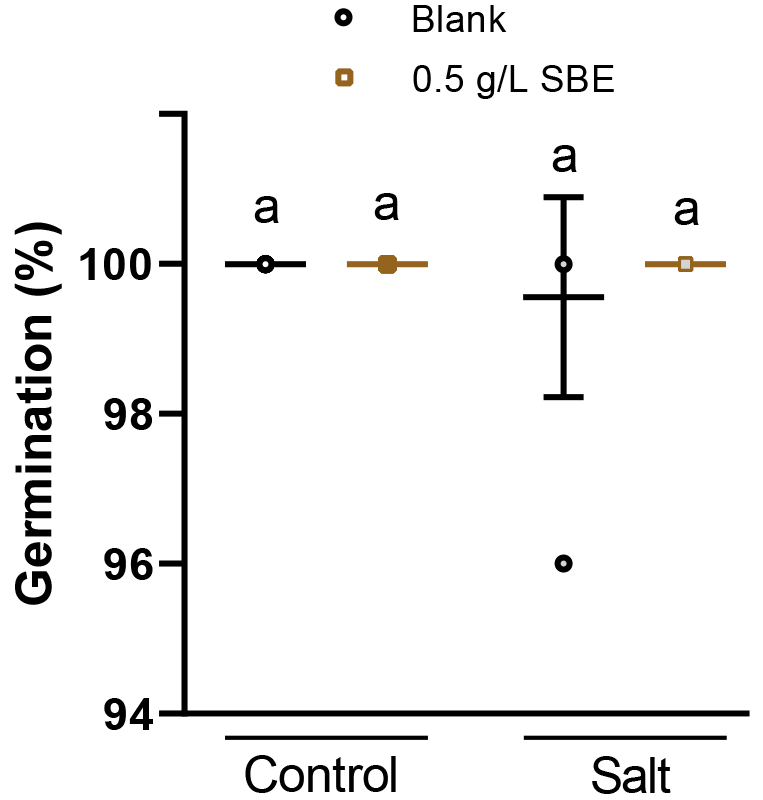

Supplement: Supplementary file 7 [file Image_5.TIF]

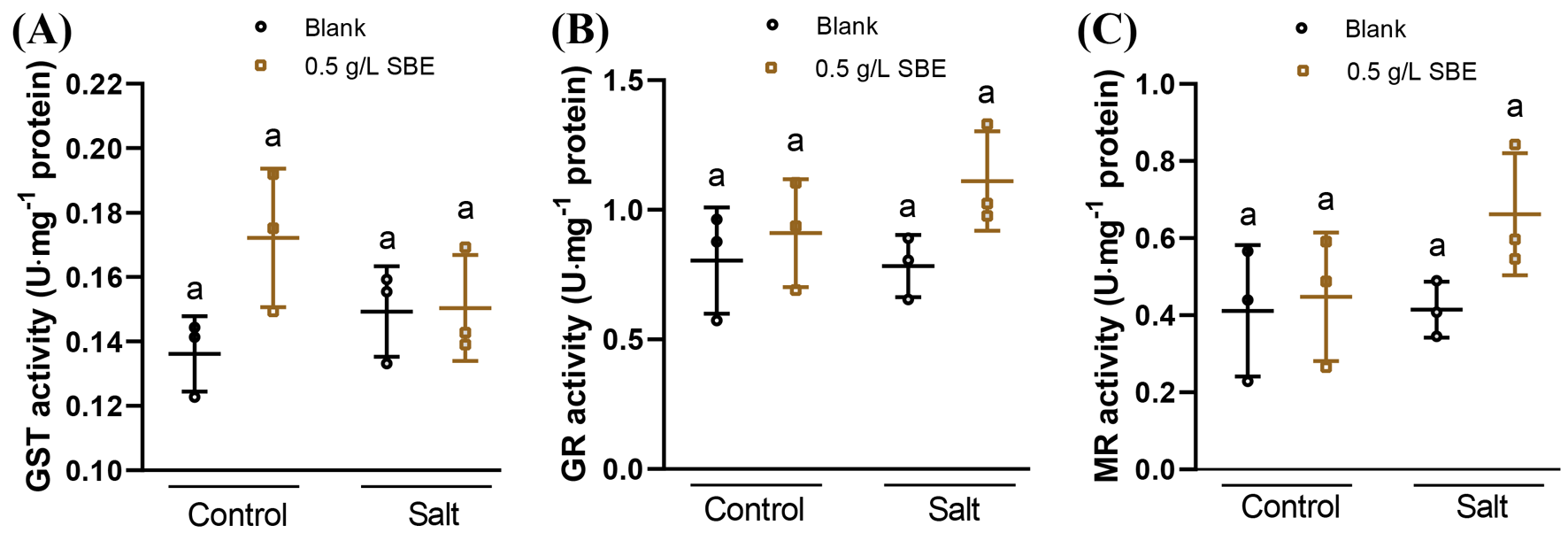

Supplement: Supplementary file 9 [file Image_7.TIF]
